# Expression of a Human Prostatic Acid Phosphatase (PAP)-IgM Fc Fusion Protein in Plants Using *In vitro* Tissue Subculture

**DOI:** 10.3389/fpls.2017.00274

**Published:** 2017-02-28

**Authors:** Yang J. Kang, Deuk-Su Kim, Soon-Chul Myung, Kisung Ko

**Affiliations:** ^1^Therapeutic Protein Engineering Laboratory, Department of Medicine, College of Medicine, Chung-Ang UniversitySeoul, South Korea; ^2^Department of Urology, College of Medicine, Chung-Ang UniversitySeoul, South Korea

**Keywords:** subculture generation, propagation, transgenic plant, recombinant protein, prostatic acid phosphatase

## Abstract

In this study, prostatic acid phosphatase (PAP), which is overexpressed in human prostate cancer cells, was cloned to be fused to the IgM constant fragment (Fc) for enhancing immunogenicity and expressed in transgenic tobacco plants. Then, the transgenic plants were propagated by *in vitro* tissue subculture. Gene insertion and expression of the recombinant PAP-IgM Fc fusion protein were confirmed in each tested the first, second, and third subculture generations (SG_1_, SG_2_, and SG_3_, respectively). Transcription levels were constantly maintained in the SG_1,_ SG_2_, and SG_3_ leaf section (top, middle, and base). The presence of the PAP-IgM Fc gene was also confirmed in each leaf section in all tested subculture generations. RNA expression was confirmed in all subculture generations using real-time PCR and quantitative real-time PCR. PAP-IgM Fc protein expression was confirmed in all leaves of the SG_1_, SG_2_, and SG_3_ recombinant transgenic plants by using quantitative western blotting and chemiluminescence immunoassays. These results demonstrate that the recombinant protein was stably expressed for several generations of *in vitro* subculture. Therefore, transgenic plants can be propagated using *in vitro* tissue subculture for the production of recombinant proteins.

## Background

Recombinant proteins are commonly produced in animal cells (Andersen and Krummen, 2002; Birch and Racher, 2006). However, production in animal cells is costly, and the cultures are susceptible to human pathogen contamination. Plants have also been effectively used as expression systems for large-scale production of recombinant proteins (Larrick and Thomas, 2001; Twyman et al., 2003; Fischer et al., 2004; Daniell et al., 2009; Ko et al., 2009; So et al., 2013; Lim et al., 2015; Park et al., 2015; Kim et al., 2016), and plant-based production has many advantages over other systems, including low biomass production costs and lack of human pathogen contamination (Twyman et al., 2003). Therefore, a wide range of recombinant biotherapeutic proteins, including industrial enzymes and new protein polymers, have been produced in plants (Ma et al., 2003). Similar to mammalian cell expression systems, plant biomass increases in an *in vitro* subculture system, and subculture can affect the protein expression level (Kim et al., 2011; Dorai and Ganguly, 2014). Although *in vitro* plant tissue subculture is an efficient method for clonal propagation, somaclonal variation generation occurred after quite prolong stage of unorganized growth, with a loss of transgene insertion and protein expression (Krishna et al., 2016). The recombinant proteins must be stably expressed in plants during *in vitro* growth so that the protein product can be extracted and purified. However, loss of the recombinant protein during plant tissue subculture is unpredictable, and sometimes, recombinant protein expression is unstable.

Prostatic acid phosphatase (PAP) is a glycoprotein that is synthesized in the epithelial cells of the prostate and is secreted into the seminal fluid (Vihko et al., 1988; McNeel et al., 2009). PAP is a prostate cancer antigen that is overexpressed by malignant prostate cell tissues and is often used as a therapeutic protein (Tarassoff et al., 2006; McNeel et al., 2009; Saif et al., 2014). In addition, due to its high expression in the prostate, PAP has been tested as a prostate cancer target antigen (Graddis et al., 2011). PAP-based peptide vaccination has been reported to induce antigen-specific T-cell responses and inhibit tumor growth in mice (Saif et al., 2014).

In this study, we examined the expression of a PAP-IgM Fc fusion protein in plant leaves from *in vitro* tissue subculture, as a vaccine candidate. The aim of this study was to determine whether PAP-IgM Fc fusion protein expression is stable over several *in vitro* subculture generations (SG_1_, SG_2_, and SG_3_).

## Materials and Methods

### Construction of the PAP-IgM Fc Gene Expression Vector

**FIGURE 1:**
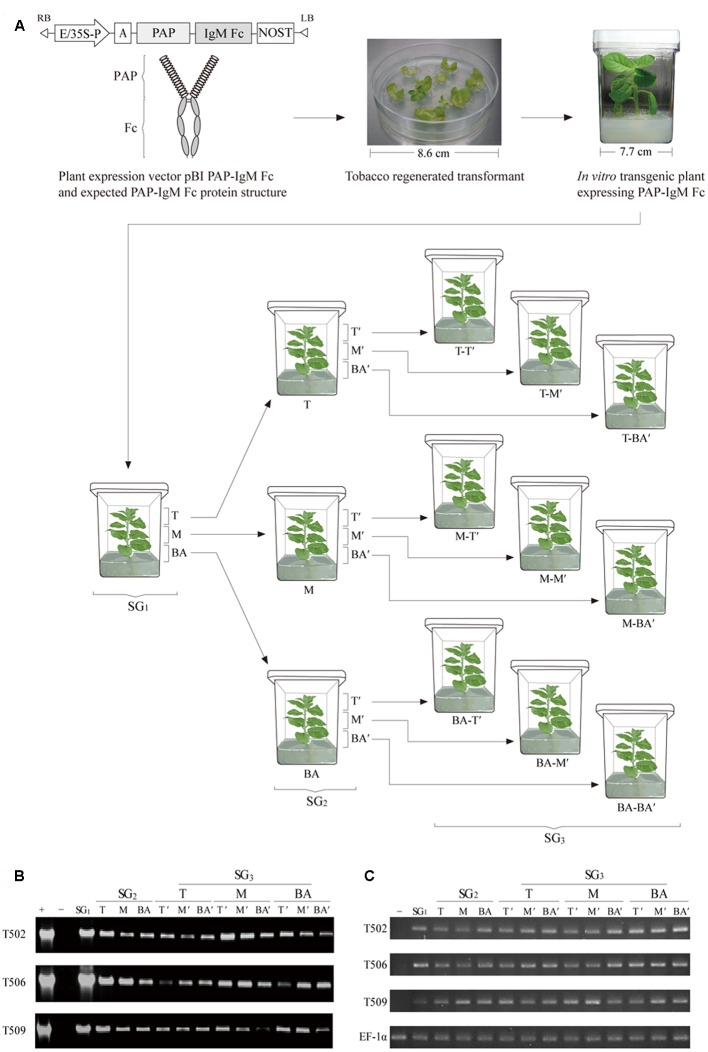
**Schematic diagram of the plant expression vector, the structure of the recombinant prostatic acid phosphatase (PAP)-IgM Fc fusion protein, plant transformation procedure, and sampling procedure for top, middle, and base leaf tissues in the various subculture generations (SG_1_, SG_2_, and SG_3_). (A)** The PAP-IgM Fc gene expression cassette in the binary pBI121 plant vector containing the cauliflower mosaic virus 35S promoter with a duplicated enhancer region (E/35S-P), the untranslated leader sequence of the tobacco etch virus, and the nopaline synthase gene terminator (NOST). Expected structure of the recombinant PAP-IgM Fc fusion protein, with a spring-shaped region (PAP) and a gray oval region (IgM Fc). A PAP-IgM Fc transgenic tobacco plantlet growing on kanamycin selection medium in a Magenta GA-7 vessel. T, top SG_1_ stem sample; M, middle SG_1_ stem sample; BA, base SG_1_ stem sample; T-T’, T of the SG_2_ stem produced from the T of the SG_1_ stem; T-M’, M of the SG_2_ stem produced from the T of the SG_1_ stem; T-BA’, BA of the SG_2_ stem produced from the T of the SG_1_ stem; M-T’, T of the SG_2_ stem produced from the M of the SG_1_ stem; M-M’, M of the SG_2_ stem produced from the M of the SG_1_ stem; M-BA’, BA of the SG_2_ stem produced from the M of the SG_1_ stem; BA-T’, T of the SG_2_ stem produced from the BA of the SG_1_ stem; BA-M’, M of the SG_2_ stem produced from the BA of the SG_1_ stem; and BA-BA’, BA of the SG_2_ stem produced from the BA of the SG_1_ stem. The circle with the dotted line indicates the part of the leaf tissue of the top portion that was harvested for analyses. **(B)** polymerase chain reaction (PCR) analysis to confirm the presence of the PAP-IgM Fc gene in tissues from subculture generations SG_1_, SG_2_, and SG_3_. PAP-IgM Fc (1,786 bp): positive control (+), pBI PAP-IgM Fc recombinant vector in DH5α competent cells, negative control (-), and non-transgenic tobacco plant (NT). The loading amount was 5 μL per sample. **(C)** Reverse transcription (RT)-PCR. Gene expressions were analyzed using RT-PCR of SG_1_, SG_2_, and SG_3_ leaf section. The negative control (-) consists of mRNA of a non-transgenic tobacco plant. The EF-1α gene was used as a housekeeping gene.

The synthetic DNA sequence encoding PAP (GenBank accession no. M34840.1) was cloned as a fusion to the Fc fragment of the human IgM μ chain (GenBank accession No. X57086.1). The PAP sequence was modified by the addition of an N-terminal extension encoding a signal peptide (MATQRRANPSSLHLITVFSLLAAVVSAEVD; Lu et al., 2012). The gene encoding PAP-IgM Fc was cloned under the control of the enhanced cauliflower mosaic virus (CaMV) 35S promoter and the tobacco etch virus 5’-leader sequence (TEV; **Figure 1A**). The PAP-IgM Fc expression cassette was subcloned into the *Hin*dIII and *Eco*RI restriction enzyme sites of the binary plant expression vector pBI121 to generate pBI PAP-IgM Fc. Then, the vector was transformed into competent *Escherichia coli* DH5α cells for amplification.

### Plant Transformation

The recombinant pBI PAP-IgM Fc vector was transferred into *Agrobacterium tumefaciens* strain LBA4404 by electroporation. Then, transgenic tobacco (*Nicotiana tabacum*) plants were generated by *Agrobacterium*-mediated transformation (Ko et al., 2003; Lu et al., 2012; So et al., 2013). Transgenic plant lines were selected on Murashige and Skoog (MS) medium [30 g⋅L^-1^ of Sucrose, 6 g⋅L^-1^ of Phyto agar, and 4.8 g⋅L^-1^ of MS B5 vitamin (Duchefa Biochemie, Haarlem, Netherlands)] containing 100 mg⋅L^-1^ kanamycin and 250 mg⋅L^-1^ cefotaxime. Transgenic plants were grown in a chamber at constant temperature (23°C) and light intensity of 50 μmol⋅m^-2^⋅s^-1^ under a long-day photoperiod (16:8 h light-dark cycle). Among the transgenic plants with high protein expression, three PAP-IgM Fc expressing transgenic lines (T502, T506, and T509) were randomly selected for *in vitro* tissue subculture.

### *In vitro* Subculture of PAP-IgM Fc Plants

Transgenic plants expressing PAP-IgM Fc were grown *in vitro* as the first generation tissue culture (SG_1_) in a Magenta GA-7 vessel (Sigma–Aldrich, St. Louis, MO, USA) for 4 weeks. Plant stems were divided into three sections [top (T), middle (M), and base (BA)]. T, M, and BA plantlet stem pieces were transplanted into new media and grown as the second-generation tissue subculture (SG_2_) for 2 weeks. This tissue subculture was repeated to generate the third generation SG_3_ (**Figure 1A**).

### Genomic DNA Extraction and PCR Analysis

Leaf tissue samples were harvested from the top portion of transgenic and non-transgenic plantlets. Genomic DNA from fresh leaf tissue was isolated using a DNA extraction kit (RBC Bioscience, Seoul, South Korea) according to the manufacturer’s protocol. The extracted genomic DNA was analyzed by polymerase chain reaction (PCR) to confirm the presence of the recombinant PAP-IgM Fc gene using the following primer pairs: forward primer 5′-GCC CTC GTT TTC AAG AAC TTG-3′ and reverse primer 5′-CGG GAT CCT CAG TAG CAG GTG CCA GCT GTG-3′. The PCR was performed with 30 cycles of 94°C for 20 s, 62°C for 20 s, and 72°C for 120 s. Genomic DNA isolated from non-transgenic plant leaves was used as the negative control, and the pBI PAP-IgM Fc gene was used as a positive control. The expected size of PAP-IgM Fc PCR product was 1,685 bp. PCR analysis was performed for more than three times.

### Real Time-PCR (RT-PCR) and Quantitative Real Time-PCR (RT-qPCR) Analyses

Leaf tissue samples were used from each top portion of transgenic plantlets. Total RNA was isolated from the leaves of transgenic plant samples (SG_1_, SG_2_, and SG_3_) using an Rneasy plant mini kit (Quagen, Valencia, CA) according to the manufacturer’s recommendations, and the isolated RNA samples were stored at -80°C. Genomic DNA removal and cDNA synthesis were performed using the Quantitect reverse transcription kit (Quagen, Valencia, CA) according to the manufacturer’s protocol. Each RNA sample was used as a template for RT-PCR and RT-qPCR analyses. RT-PCR and RT-qPCR reactions were performed using the Maxime PCR premix kit (Intron Biotechnology, Seoul, South Korea) and Rotor-Gene SYBR Green PCR kit (Quagen, Valencia, CA), respectively. PAP-IgM Fc primers were as follows: 5′-CTC ATG CTA CCT GGT TGC AG-3′; forward, 5′-GGT GGG ACG AAG ACG CTC A-3′. Quantitative RT-PCR was analyzed using real-time PCR machine (Rotor-Gene Q, Quagen) with the following cycling parameters: 5 min at 95°C, 5 s at 95°C, 10 s at 60°C, and 40 cycles of 5 s at 95°C and 10 s at 60°C. The elongation factor 1-α (EF-1α) gene was used as a housekeeping gene. RT PCR and RT-qPCR analyses were performed for 3 times.

### Quantitative Immunoblot Analysis

**FIGURE 2:**
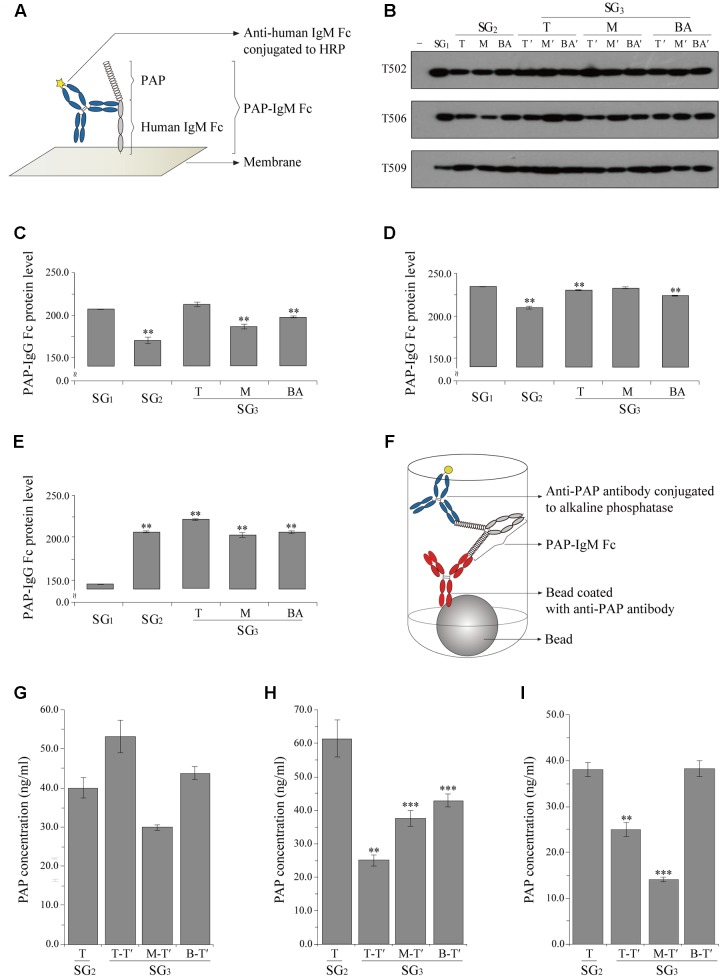
**Densitometry analysis of PAP-IgM Fc protein expression levels in the tissues of plantlets from subculture generations SG_1_, SG_2_, and SG_3_ (A–E)** and chemiluminescence immunoassay (CLIA) to confirm PAP-IgM Fc protein expression in various leaf tissues from subculture (T of SG_2_ and T-T, M-T’, and BA-T’ of SG_3_ protein expression SG_3_ **(F–I)**. **(A)** Illustration showing the interaction between PAP-IgM Fc and the anti-human IgM antibody conjugated to horseradish peroxidase (HRP) in the western blot. **(B)** Western blot analysis to confirm PAP-IgM Fc protein expression in top (T), middle (M), and base (BA) stem tissues from different subculture generations (SG_1_, SG_2_, and SG_3_). Negative control (-) and non-transgenic plant (NT). PAP-IgM Fc (83 kDa) was detected with HRP-conjugated goat anti-human IgM (μ chain) immunoglobulin. Black arrowheads indicate the PAP-IgM Fc protein band (83 kDa). Expression levels of PAP-IgM Fc in T502 **(C)**, T506 **(D)**, and T509 **(E)** leaf tissues were assessed by western blotting with an anti-human IgM (μ chain) immunoglobulin conjugated to HRP. Protein expression rates in the three PAP-IgM Fc transgenic lines were calculated based on the protein band density in the western blot analysis. **(F)** Illustration of the double sandwich CLIA. The interaction between PAP-IgM Fc and the anti-human IgM antibody conjugated to alkaline phosphatase. The expression in the transgenic lines T502 **(G)**, T506 **(H)**, and T509 **(I)** PAP-IgM Fc proteins in transgenic plants from the top of SG_2_ and SG_3_. Data are the mean and standard error (^∗∗∗^*p* < 0.01, ^∗∗^*p* < 0.05, *t*-test).

The PAP-IgM Fc protein expression level in transgenic lines T502, T506, and T509 was investigated using immunoblot analysis. Leaf samples (100 mg) were harvested from the plantlet top and homogenized in 300 μL of 1 × PBS (137 mM NaCl, 10 mM Na_2_HPO_4_, 2.7 mM KCl, and 2 mM KH_2_PO_4_) to extract total soluble proteins. PAP-IgM Fc leaf extracts were boiled with 5 × protein loading buffer (1 M Tris-HCl, 50% glycerol, 10% SDS, 5% 2-mercaptoethanol, and 0.1% bromophenol blue) for 10 min and cooled for 2 min. Total soluble proteins were separated by 10% SDS-PAGE and transferred to a nitrocellulose membrane (Millipore, Billerica, MA, USA). Membranes were incubated with 5% skim milk (Sigma, St. Louis, MO, USA) in 1 × PBS buffer at 4°C overnight. Blots were incubated with a goat anti-human IgM (μ chain) antibody conjugated to horseradish peroxidase (Abcam Inc., Cambridge, MA, USA) diluted 1:5,000 in 1 × PBS solution for 2 h at room temperature (**Figure 2A**). After three 10-min washes, proteins were detected with the SuperSignal West Pico Chemiluminescent Substrate (Thermo Scientific, Rockford, IL, USA) and visualized by exposing the membrane to X-ray film (Fuji, Tokyo, Japan). Leaf tissue extract from non-transgenic plants was used as a negative control. Detected protein bands were digitized as an electronic image, and band intensity was measured using ImageJ software (National Institutes of Health, Bethesda, MD, USA). Immunoblot analysis was performed for more than three times.

### Chemiluminescence Immunoassay

Expression of PAP-IgM Fc in plant leaf tissue was analyzed using a chemiluminescence immunoassay (CLIA). Leaf samples from PAP-IgM Fc transgenic plants were harvested from the plantlet top (T of SG_2_ and T-T’, M-T’, and BA-T’ of SG_3_; **Figure 1A**) and homogenized in 300 μL of 1 × PBS (137 mM NaCl, 10 mM Na_2_HPO_4_, 2.7 mM KCl, and 2 mM KH_2_PO_4_) for total soluble protein extraction. All samples were diluted 1:100 in 1 × PBS solution. A cuvette containing the anti-PAP coated beads (Siemens Healthcare Diagnostics Inc., Llanberis, UK) was incubated with PAP-IgM Fc and an anti-PAP antibody conjugated to alkaline phosphatase for 30 min at 37°C (**Figure 2F**). After the antibodies were removed and the beads were washed in deionized distilled water as a centrifugal washing buffer, the PAP-IgM Fc proteins were detected using the chemiluminescent substrate. The samples were analyzed on an IMMULITE 2000 xpi (Siemens Healthcare Diagnostic Inc., Flanders, NJ, USA). CLIA was performed for more than three times.

### Statistical Analysis

All values are shown as the mean ± SD. Recombinant PAP-IgM Fc protein expression in transgenic plant leaves was compared by using the unpaired *t*-test, and *p* values less than 0.01 (^∗∗∗^) or 0.05 (^∗∗^) were considered statistically significant. Statistical significance was assessed using Excel (Microsoft Office Excel 2013; Microsoft Corporation, Redmond, WA, USA).

## Results

### Confirmation of PAP-IgM Fc Gene Presence

Transgenic tobacco plant lines were obtained by *Agrobacterium*-mediated transformation with plant expression vectors carrying PAP-IgM Fc. Genomic DNA extracted from three transgenic lines (T502, T506, and T509) was analyzed by PCR to confirm insertion of the PAP-IgM Fc transgene. The PAP-IgM Fc gene was detected in the top leaves of each subculture generation (SG_1_, SG_2_, and SG_3_) obtained from the T, M, and BA stems harvested from tobacco transgenic plants (SG_1_). The size of the PAP-IgM Fc PCR product in the genome of the transgenic plants was 1,685 bp (**Figure 1B**). No PAP-IgM Fc PCR product was detected in non-transgenic plants.

### Gene Transcription Level Analysis of PAP-IgM Fc in All Subculture Generations (SG_1_, SG_2_, and SG_3_)

In real-time PCR and quantitative real-time PCR, the relative transcription level was observed in the top portions of all subculture generations (SG_1_, SG_2_, and SG_3_) leaf tissues (Supplementary Figure S1). The transgene was not detected in non-transgenic plant (**Figure 1C**). RT-PCR product of the expected size of PAP-IgM Fc was detected in all samples of transgenic plants (T502, T506, and T509; **Figure 1C**). In RT-qPCR, the PAP-IgM Fc transcriptional levels were consistently higher than 0.9 level in SG_1_, SG_2_, and SG_3_ leaf section (T, M, and BA; Supplementary Figure S1). Non-transgenic plant showed no PAP-IgM Fc signal.

### Confirmation of PAP-IgM Fc Protein Expression

PAP-IgM Fc protein expression in leaf samples from transgenic plants was analyzed by western blotting. All transgenic plant lines harboring the PAP-IgM Fc transgene showed a protein band of approximately 83 kDa (**Figure 2B**). Recombinant PAP-IgM Fc fusion proteins were expressed in all subculture generations (SG_1_, SG_2,_ and SG_3_) obtained from the T, M, and BA stems of SG_1_ transgenic plants (**Figure 2B**). No PAP-IgM Fc protein band was detected in non-transgenic plants, which were used as a negative control (**Figure 2B**).

### Densitometry Analysis of PAP-IgM Fc Protein Expression Levels in SG_1_, SG_2_, and SG_3_

Quantitative western blot analysis was performed to determine the expression level of the recombinant PAP-IgM Fc fusion protein in the top leaves of SG_1_, SG_2_, and SG_3_ (**Figure 2B**). The intensity of the PAP-IgM Fc fusion protein bands in the top leaf samples was quantified using Image J software. Although the PAP-IgM Fc recombinant protein expression level differed slightly among the SG_1_, SG_2_, and SG_3_ samples, all leaf samples showed stable expression of the recombinant protein (**Figures 2C–E**).

### Quantitative CLIA of PAP-IgM Fc in Transgenic Plant Leaves

A CLIA was performed to quantify the expression of recombinant PAP-IgM Fc protein in the transgenic leaves of SG_2_ and SG_3_ plants. In T502, PAP-IgM Fc expression levels in each leaf position were slightly different. However, the mean values at each position in SG_3_ were not significantly different (**Figure 2G**). In T506, the PAP-IgM Fc expression levels were significantly different at each leaf position, and the mean values at each position in SG_3_ were significantly different (**Figure 2H**). In T509, the PAP-IgM Fc expression levels in SG_2_, T-T’ of SG_3_, and M-T- of SG_3_ differed. However, the mean values at all positions were not significantly different (**Figure 2I**). Overall, the expression of the recombinant PAP-IgM Fc fusion protein was maintained without loss in all *in vitro* SG samples (**Figures 2G–I**).

## Discussion

This study demonstrated that the production of different subculture generations of transgenic plants did not affect the expression of the recombinant PAP-IgM Fc fusion protein. The fusion of antigenic protein to IgM Fc can assembled to form large quaternary circular protein enhancing immunogenicity (David et al., 2011). The PAP is a PAP glycoprotein fused to the Fc fragment of the human IgM immunoglobulin. The PAP-IgM Fc gene was constitutively expressed under the control of the enhanced CaMV 35S promoter (Lu et al., 2012; Lim et al., 2015). The N-terminus of PAP was fused to a signal peptide.

In this study, we hypothesized that PAP-IgM Fc expression in transgenic plants would be stable through several generations produced by *in vitro* tissue subculture. With this in mind, each cultured stem section (T, M, and BA) was transplanted and grown to investigate the stability of the transgene insertion and transgenic protein expression. PCR analysis confirmed that the recombinant PAP-IgM Fc gene insertion was stable in all tested subculture generations (SG_1_, SG_2_, and SG_3_). *In vitro* subcultured transgenic plants may lose the recombinant gene during propagation (Fladung and Kumar, 2002; Zeng et al., 2010; Martinelli et al., 2015). Loss of recombinant genes may occur because they are not strongly attached to the genomic DNA of plant cells (Doran, 2006). However, in this study, stable insertion of the PAP-IgM Fc gene was confirmed in all subculture generations. RT-PCR and RT-qPCR analysis were applied to investigate whether the mRNA levels of the PAP-IgM Fc gene expression at all subculture generations (SG_1_, SG_2_, and SG_3_). The mRNA levels slightly differed at each subculture generations. However, transcription levels in all transgenic plants were constantly maintained at each subculture generation (Supplementary Figure S1). These results indicate that the PAP-IgM Fc transgene was not deleted during tissue propagation.

Immunoblot analysis and CLIA were conducted to investigate whether the PAP-IgM Fc fusion protein was expressed in the different subculture generations. The protein expression levels slightly differed at leaf tissue positions among SG_1_, SG_2_, and SG_3_. Such of the PAP-IgM Fc protein level is due to leaf tissue position and developmental stage, at which water content and plant cell number might be variable (Streatfield, 2007; Lim et al., 2015). Thus, in the future normalization for protein level quantification should be optimized, which can precisely calculate amount of recombinant protein per amount of total soluble protein in plant tissue (Alkanaimsh et al., 2016). In addition, the protein level might be fluctuated by variation factors, which are total soluble protein levels to be hardly controlled because of low ratio between leaf tissue material weight and homogenizing buffer volume in this study. However, all leaf samples harvested from the top portion of the plants showed stable expression of the PAP-IgM Fc recombinant protein. In addition, the PAP-IgM Fc recombinant protein was expressed without degradation in a consistent pattern in all subculture generations (SG_1_, SG_2_, and SG_3_). The PAP expression levels in each plant sample, as quantified by western blotting, were correlated with the PAP levels detected by CLIA (data not shown). This shows that both these quantitative methods are reliable for confirming PAP expression in transgenic plant tissues.

Plant expression systems are advantageous both in terms of safety and production cost, when compared to conventional cell systems, such as yeast, mammalian, and insect cells (Twyman et al., 2003; Ko, 2014). In animal cell systems, loss of recombinant protein expression often occurs during subculture due to deletion of the recombinant transgenes (Heller-Harrison et al., 2009; Kim et al., 2011; Dorai and Ganguly, 2014).

The ultimate purpose of *in vitro* plant tissue subculture is the mass propagation of transgenic plants. However, somatic variations that occur during *in vitro* subculture can alter the genetic components and protein expression. Therefore, expression of the recombinant protein in the transgenic plants must be confirmed through *in vitro* subculture.

Taken together, the transgenic plants obtained through *in vitro* tissue subculture showed that the transgene and transgenic protein expression were stable. Thus, transgenic plants can be used to produce highly valuable therapeutic recombinant proteins via *in vitro* subculture. PAP is highly expressed in human prostate cancer cells, which can be a promising vaccine candidate. In addition, PAP itself can be used as one of prostate cancer indicators in medical diagnosis. Thus, plant-derived recombinant PAP-IgM Fc fusion protein can be highly useful in both research medical and purposes.

## Author Contributions

YK contributed for acquisition of data and writing of manuscript. D-SK contributed for acquisition of data and interpreted data. S-CM analyzed and interpreted data, and KK made substantial contributions to conception and design of the study.

## Conflict of Interest Statement

The authors declare that the research was conducted in the absence of any commercial or financial relationships that could be construed as a potential conflict of interest.
