# Supplementary material for: Expression of a Human Prostatic Acid Phosphatase (PAP)-IgM Fc Fusion Protein in Plants Using In vitro Tissue Subculture
Source: Front Plant Sci. 2017 Feb 28;8:274. doi: 10.3389/fpls.2017.00274 (PMC5329016; doi:10.3389/fpls.2017.00274)
Supplement: Supplementary file 1 [file Data_Sheet_1.DOCX]

***Supplementary Figure***


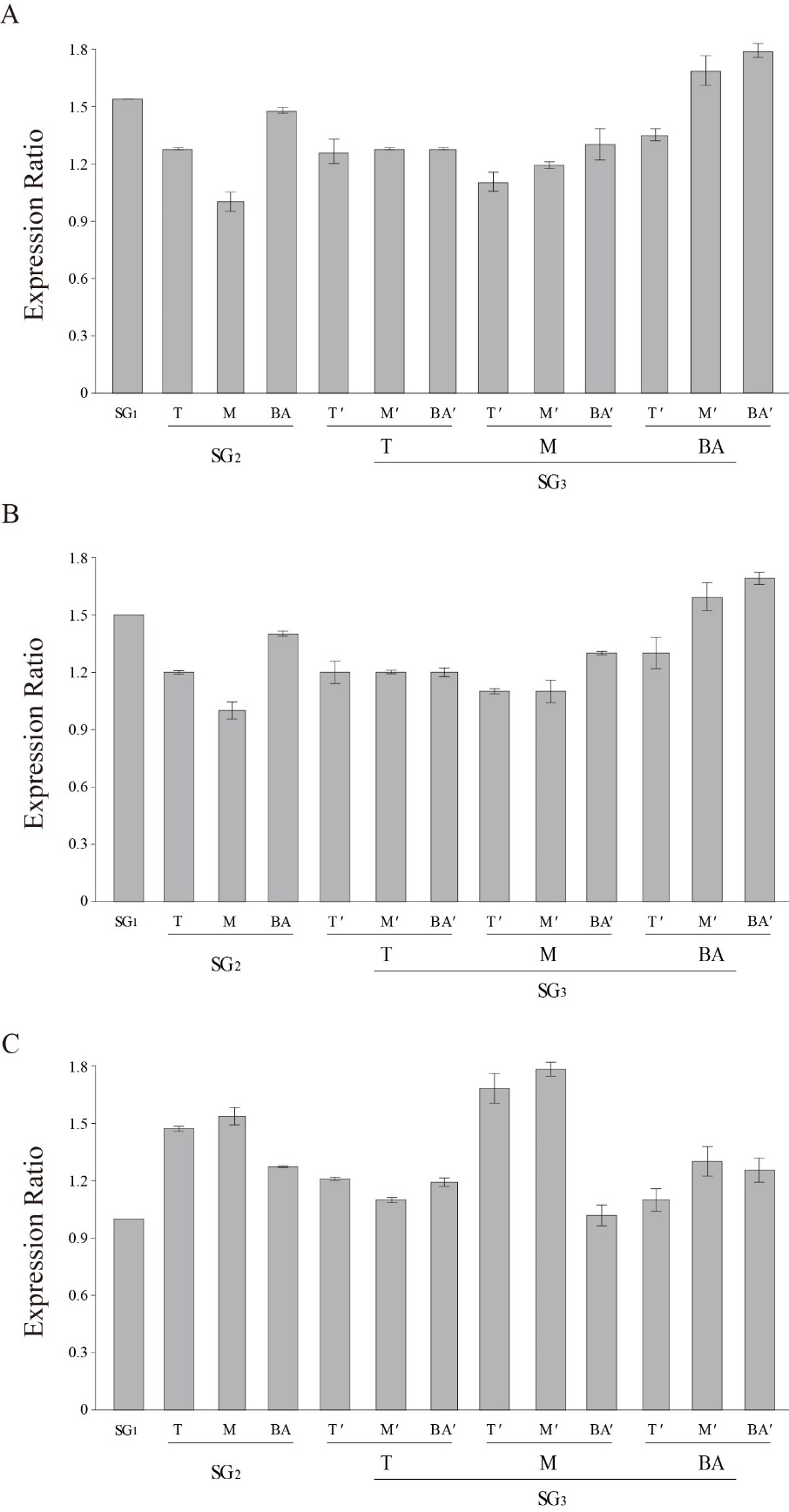


**Supplementary Figure S1.** RNA expression levels of PAP-IgM Fc by quantitative RT-PCR. Relative expression levels of PAP-IgM Fc with transgenic line [T502 (A), T506 (B), T509 (C)] at each subculture generation of plant leaf **were estimated** by quantitative RT-PCR using total RNA.
